# Melatonin overcomes MCR-mediated colistin resistance in Gram-negative pathogens

**DOI:** 10.7150/thno.45951

**Published:** 2020-08-29

**Authors:** Yuan Liu, Yuqian Jia, Kangni Yang, Ziwen Tong, Jingru Shi, Ruichao Li, Xia Xiao, Wenkai Ren, Rüdiger Hardeland, Russel J. Reiter, Zhiqiang Wang

**Affiliations:** 1 College of Veterinary Medicine, Yangzhou University, Yangzhou, Jiangsu 225009, China.; 2 Jiangsu Co-innovation Center for Prevention and Control of Important Animal Infectious Diseases and Zoonoses, Joint International Research Laboratory of Agriculture and Agri-Product Safety, the Ministry of Education of China, Yangzhou University, Yangzhou, Jiangsu 225009, China.; 3 Institute of Comparative Medicine, Yangzhou University, Yangzhou, Jiangsu 225009, China.; 4 State key Laboratory for Conservation and Utilization of Subtropical Agro-Bioresources, Guangdong Laboratory of Lingnan Modern Agriculture, National Engineering Research Center for Breeding Swine Industry, Guangdong Provincial Key Laboratory of Animal Nutrition Control, College of Animal Science, South China Agricultural University, Guangzhou 510642, China.; 5 Johann Friedrich Blumenbach Institute of Zoology and Anthropology, University of Göttingen, 37073 Göttingen, Germany.; 6 Department of Cellular and Structural Biology, University of Texas Health Science Center, San Antonio, 78229 TX, USA.

**Keywords:** antibiotic adjuvant, bacterial infections, colistin, gram-negative pathogen, melatonin

## Abstract

**Background:** Emergence, prevalence and widely spread of plasmid-mediated colistin resistance in Enterobacteriaceae strongly impairs the clinical efficacy of colistin against life-threatening bacterial infections. Combinations of antibiotics and FDA-approved non-antibiotic agents represent a promising means to address the widespread emergence of antibiotic-resistant pathogens.

**Methods:** Herein, we investigated the synergistic activity between melatonin and antibiotics against MCR (mobilized colistin resistance)-positive Gram-negative pathogens through checkerboard assay and time-killing curve. Molecular mechanisms underlying its mode of action were elucidated. Finally, we assessed the *in vivo* efficacy of melatonin in combination with colistin against drug-resistant Gram-negative bacteria.

**Results:** Melatonin, which has been approved for treating sleep disturbances and circadian disorders, substantially potentiates the activity of three antibiotics, particularly colistin, against MCR-expressing pathogens without enhancing its toxicity. This is evidence that the combination of colistin with melatonin enhances bacterial outer membrane permeability, promotes oxidative damage and inhibits the effect of efflux pumps. In three animal models infected by *mcr-1*-carrying *E. coli*, melatonin dramatically rescues colistin efficacy.

**Conclusion:** Our findings revealed that melatonin serves as a promising colistin adjuvant against MCR-positive Gram-negative pathogens.

## Introduction

Antibiotic resistance is a growing problem that threatens the conventional regimens to treat bacterial infectious diseases 1, 2. It has been predicted that the antibiotic resistant bacteria would kill 10 million lives per year and lead to 100 trillion USD of economic loss worldwide by 2050 3. Colistin, a nonribosomal peptide antibiotic, is one of last-resort antibiotics against multidrug-resistant Gram-negative pathogens, particularly for carbapenem-resistant Enterobacteriaceae 4, 5. The bactericidal activity of colistin is mainly dependent on the disruption of membrane permeability and leakage of bacterial components through the electrostatic interaction between positively-charged residues of colistin and negatively charged lipid A moieties of lipopolysaccharides (LPS) anchored to the bacterial outer membrane 6. However, the mobilized colistin resistance gene (*mcr-1*) and its variants that encode phosphoethanolamine transferases reduce the negative charge of lipid A and confer a substantial resistance to colistin 7, 8. More problematically, the *mcr-1* has already spread to over 40 countries/regions covering five of seven continents 9. Therefore, there is an urgent need to identify novel strategies to overcome MCR-mediated acquired colistin resistance in Gram-negative pathogens.

Compared with the time and money-consuming development of novel antibiotics, antibiotic adjuvant strategy offers a more cost-effective approach by preventing bacterial resistance or enhancing antibiotic modes of action 10-12. For example, inhibitors of β-lactamases such as clavulanic acid is the most clinically successful adjuvant to date 13. In addition, a fungus-derived product aspergillomarasmine A rescues meropenem activity by suppressing metallo-β-lactamase (MBLs) activity 14. Cholesterol lowering drugs such as statins disassemble bacterial membrane microdomains, and restore MRSA susceptibility to penicillin 15. The anti-protozoal drug pentamidine potentiates hydrophobic antibiotics activity against Gram-negative pathogens through disrupting the bacterial outer membrane 16. These examples inspired us to look for colistin adjuvants from the FDA-approved compounds. Thus far, several strategies have been reported to partially restore colistin activity 17-21. For instance, pterostilbene is a natural product obtained from fresh leaves or fruits that enhances the therapeutic effect of polymyxins 17, but the underlying mechanism is unclear. In addition, the combination of colistin with Gram-positive antibiotics such as rifampicin also contribute to overcoming *mcr-1* mediated colistin resistance 18. However, this combination efficacy is highly dependent on the antibacterial effect of Gram-positive antibiotics on membrane-disrupted Gram-negative bacteria, and may be ineffective against multidrug-resistant pathogens. Despite these ongoing efforts, no colistin adjuvants have been tested in human clinical trials so far due to practical and technical limitations. Thus, safer and more effective colistin adjuvants for resistant bacteria are urgently required.

Melatonin (*N*-acetyl-5-methoxytryptamine) is a neurohormone that transmits the information “darkness” and contributes to the synchronization of circadian oscillators 22. Moreover, it is involved in numerous other physiological processes 23 such as regulation of blood pressure 24 and core body temperature 25, suppression of oncogenesis 26, and immune function 27. Melatonin is produced by the pineal gland and possibly all extra-pineal organs including the skin, retina, cerebellum, kidneys, liver, pancreas, and ovaries, as well as in plants and other phototrophic organisms 28. Although multiple beneficial effects of melatonin have been uncovered, its potential application in treatment of pathogenic bacteria has not been fully explored. Herein, we focused on the potency of melatonin as a novel antibiotic adjuvant. Interestingly, we found that melatonin effectively reverses MCR-mediated colistin resistance both *in vivo* and *in vitro* through multiple modes of action. The discovery of melatonin as a new and safe colistin adjuvant provides a therapeutic regimen for combating Gram-negative bacteria infections.

## Materials and Methods

### Bacteria and reagents

Strains used in this study are listed in **Table S1**. *Escherichia coli* MG1655 deleted mutants were obtained through homologous recombination mediated by suicide plasmid pLP12 and confirmed by PCR analysis. Isolation and detection of clinical isolates were performed based on previous study 29. Broth suspension of samples were inoculated onto MacConkey agar plates containing 2 μg/mL colistin. Positive colonies were confirmed for the *mcr-1* gene by PCR analysis. Unless otherwise noted, strains were grown in Mueller-Hinton Broth (MHB) or on MH agar (MHA) plates. CHO and HEK293T cells were grown in DMEM (Gibco, MA, USA) supplemented with 10% heat-inactivated FBS (Invitrogen, CA, USA). Melatonin was purchased from Sigma-Aldrich (MO, USA). Antibiotics were obtained from China Institute of Veterinary Drug Control and other chemical reagents were purchased from Aladdin (Shanghai, China).

### Antibacterial test

MICs of compounds were measured using the standard broth micro-dilution method, according to the CLSI 2016 guideline 30 and previous report 31. All drugs were two-fold diluted in MHB and equally mixed with bacterial suspensions in a 96-well microtiter plate (Corning, New York, USA). MIC values were defined as the lowest concentrations of drugs with no visible growth of bacteria after 18 h incubation at 37 °C.

### Checkerboard assays

Synergistic activity of antibiotics and melatonin was evaluated by checkerboard assays with two-fold serially dilution of drugs (8 × 8 matrix). After 18 h co-incubation with bacterial suspension (1.5 × 10^6^ CFUs/well), the absorbance of bacterial culture at 600 nm was measured by Microplate reader. Two biological replicates were performed for each combination and the means were used for FIC index (FICI) calculation according to the formula as follows 32:

FIC index = FICI_a_ + FICI_b_ = MIC_ab_ / MIC_a_ + MIC_ba_ / MIC_b._

MIC_a_ is the MIC of compound A alone; MIC_ab_ is the MIC of compound A in combination with compound B; MIC_b_ is the MIC of compound B alone; MIC_ba_ is the MIC of compound B in combination with compound A. Synergy is defined as an FIC index of ≤ 0.5.

### Time-dependent killing

Overnight *E. coli* B2 were diluted 1/1,000 in MHB, and incubated for 4 h (exponential phase) or 8 h (stationary phase) at 37 °C. Bacteria were then treated with melatonin (1 mg/mL) and colistin (2 μg/mL) alone, or their combination for 24 h. At intervals, 100 μL aliquots were removed, centrifuged and resuspended in 100 μL sterile PBS. Subsequently, ten-fold serially diluted suspensions were plated on MHA plates and incubated overnight at 37 °C. Bacterial colonies were counted and the primary CFUs/mL was calculated.

*E. coli* MG1655 and its four deletion mutants at stationary phase were challenged with the combination of melatonin (1 mg/mL) and (0.0625 μg/mL) for 8 h. At intervals, the colonies (CFUs/mL) were counted and calculated. All experiments were performed with at least three biological replicates.

### Safety assessment

Hemolytic activity of colistin in the presence of melatonin was evaluated based on previous reports 33, 34. 8% sheep blood cells were treated with colistin (16 to 128 μg/mL) and/or melatonin (0 to 1,000 μg/mL) for 1 h. Triton X-100 (0.2%) was used as a positive control. After incubation, the absorbance of supernatant at 576 nm was measured and hemolysis rate was calculated by comparing with positive control.

Cytotoxicity on CHO and HEK293T cells was determined using the water-soluble tetrazolium salt-1 (WST-1, Roche, Switzerland) assay 35. Colistin (0 to 128 μg/mL) with melatonin (0 to 1,000 μg/mL) and 1 × 10^4^ cells were simultaneously added into 96-well plates and cultured at 37 °C for 24 h. Then, the absorbance of cell culture at 450 nm was measured and corresponding cytotoxicity was calculated.

### Transcriptomic analysis

*E. coli* B2 were grown in MHB to the early-exponential phase, and treated with colistin (40 μg/mL) alone or in combination of melatonin (1 mg/mL) for 4 h. Total RNA of samples was extracted by an EASYspin Plus kit (Aidlab, Beijing, China) and quantified by using a Nanodrop spectrophotometer (Thermo Scientific, MA, USA), and sequenced by using the Illumina Hiseq 2000 system (Majorbio, Shanghai, China). Library construction of purified mRNA was conducted with Illumina Truseq RNA sample prep Kit according to the manufacturer's protocol. After amplification by bridge PCR with Illumina Truseq PE Cluster Kit v3-cBot-HS on cBot (Illumina), samples were sequenced by using Hiseq2000 Truseq SBS Kit v3-HS (200 cycles) (Illumina) with the read length as 2 × 100 (PE100). Raw sequencing reads were filtrated and mapped against the reference genome of *E. coli* K-12. Differentially-expressed genes were identified by using the FPKM (Fragments Per Kilobase of transcript per Million mapped reads) method with *p*-values ≤ 0.05 and fold change (FC) values ≥ 2 (log2 FC ≥ 1 or log2 FC ≤ -1). Differences between these two treatments were analyzed by Cuffdiff program (http://cufflinks.cbcb.umd.edu/).

### RT-PCR analysis

*E. coli* B2 were grown to early-exponential phase, and incubated with colistin (40 μg/mL) alone or in combination of melatonin (1 mg/mL) for 4 h. Then, total RNA was extracted and quantified by the ratio of absorbance (260 nm/280 nm). Reverse transcription of 1 μg extracted RNA was performed using the PrimeScript™ RT reagent Kit with gDNA Eraser (Takara, Dalian, China) following the manufacturer's protocol. RT-PCR analysis was performed by 7500 Fast Real-Time PCR System (Applied Biosystem, CA, USA) using the TB Green qPCR Kit (Takara) with the optimized primers **(Table S4)**. Relative quantitative method was applied to calculate the fold changes of mRNA expression relative to the reference genes (16S rRNA) in *E. coli*.

### Biochemical factors analysis

Pretreatments of biochemical assays were performed using similar protocols as follows. Overnight *E. coli* B2 were washed, suspended in 5 mM HEPES (pH 7.0, plus 5 mM glucose) with an OD_600_ of 0.5, and incubated with fluorescent dyes for 30 min. Then, 190 μL of probed-cells were mixed with 10 μL of melatonin alone (0 to 1,000 μg/mL), or colistin (0 to 64 μg/mL) without or with melatonin (500 μg/mL) in a 96-well plate. After incubation at 37 °C for 1 hour, fluorescence intensity or absorbance or luminescence was measured by an Infinite M200 Microplate reader (Tecan).

### Outer membrane permeability

1-*N*-phenylnaphthylamine (NPN) (10 μm) 36 with the excitation wavelength of 350 nm and emission wavelength of 420 nm was used to evaluate the outer membrane permeability.

### Cell membrane integrity

Fluorescence intensity of 10 nM propidium iodide (PI)-labeled cells in the presence of increasing drugs was measured with the excitation wavelength of 535 nm and emission wavelength of 615 nm.

### Membrane depolarization

3, 3-dipropylthiadicarbocyanine iodide (DiSC_3_(5), 0.5 μM) was applied to determine the membrane potential 37. Dissipated membrane potential of *E. coli* B2 was measured with the excitation wavelength of 622 nm and emission wavelength of 670 nm.

### Total ROS and H_2_O_2_

2′,7′-dichlorodihydrofluorescein diacetate (DCFH-DA, 10 μM) was applied to monitor levels of ROS in *E. coli* B2 38, following the manufacturer's instruction (Beyotime). Fluorescence intensity was measured with the excitation wavelength of 488 nm and emission wavelength of 525 nm. In addition, production of H_2_O_2_ in* E. coli* B2, induced by melatonin in the absence and presence of colistin, was determined by a Hydrogen Peroxide Assay Kit (Beyotime, China). After incubation for one hour, the absorbance of lysis buffer at 570 nm was measured.

### Intracellular ATP

Intracellular ATP levels of *E. coli* B2 were determined using an Enhanced ATP Assay Kit (Beyotime, China). Overnight* E. coli* B2 were washed and resuspended to obtain an OD_600_ of 0.5. After treating with various concentrations of colistin alone or in combination with melatonin (500 μg/mL) for 1 h, bacterial cultures were centrifuged and the supernatant was removed. Bacterial precipitates were lysed by lysozyme, and the supernatant was prepared for intracellular ATP levels measurement. Detecting solution was added to a 96-well plate and incubated at room temperature for 5 min. Subsequently, the luminescence of supernatants was monitored by Infinite M200 Microplate reader (Tecan). Intracellular ATP levels in *E. coli* were calculated from the luminescence signals.

### SOD activity

Intracellular superoxide dismutase (SOD) activity of *E. coli* B2 treated with melatonin, colistin or their combination was measured using the Total Superoxide Dismutase Assay Kit with WST-8 (S0101, Beyotime, China).

### Animal studies

6-8-week-old female BALB/c mice were obtained from Comparative Medicine Centre of Yangzhou University (Jiangsu, China). Mice were adapted for one week prior to infection. Mouse studies were performed in accordance with the relevant guidelines and regulations (ID: SCXK-2017-0007). The laboratory animal usage license number is SCXK-2017-0044, certified by Jiangsu Association for Science and Technology.

### Pharmacokinetic analysis

BALB/c female mice were intraperitoneally injected with a single dose of colistin (10 mg/kg) and melatonin (50 mg/kg). Plasma samples were taken from three mice at each time point (5 min, 10 min, 15 min, 30 min, 1 h, 2 h, 4 h, 8 h, 12 h and 24 h). Plasma (100 μL) was mixed with acetonitrile (300 μL), vigorously vortexed and centrifuged at 12,000 rpm for 10 min. The precipitate was re-extracted with acetonitrile (100 μL). Combined supernatants were filtered through a 0.22 μm filter membrane before LC-MS/MS analysis. Colistin and melatonin concentrations in supernatants were determined by AB SCIEX 6500 QTRAP™ mass spectrometer (Applied Biosystems, CA, USA) with positive ionization multiple reaction monitoring (MRM) mode **(Table S5)**. 0.1% formic acid in water and 0.1% formic acid in acetonitrile were used as mobile phase. Limit of detection (LOD), limit of quantitation (LOQ), recoveries and intra-day relative standard deviation (RSD) of detection method are presented in **Tables S6** and **S7**. Pharmacokinetic parameters were performed using a non-compartmental analysis model by WinNonlin 6.4 software.

### *Galleria mellonella* infection model

*Galleria mellonella* larvae (Huiyude Biotech Company, Tianjin, China) were randomly divided into four groups (n = 10 per group) and infected with *E. coli* B2 suspension (10 µL, 1.0 × 10^5^ CFUs per larvae) at the right posterior gastropoda. At one-hour post-infection, *Galleria mellonella* larvae were injected with PBS, colistin (10 mg/kg), melatonin (50 mg/kg), or the combination of colistin with melatonin (10 + 50 mg/kg) at left posterior gastropoda. Survival rates of *Galleria mellonella* larvae were recorded for 5 days.

### Mouse peritonitis-sepsis infection model

Female BALB/c mice (n = 8 or 9 per group) were intraperitoneally infected with a dose of 3.0 × 10^8^ CFUs* E. coli* B2 suspension. At one-hour post-infection, mice were treated with a single dose of colistin (10 mg/kg), melatonin (50 mg/kg), or combinations of colistin plus melatonin (5 + 50 mg/kg, 10 + 20 mg/kg or 10 + 50 mg/kg) via intraperitoneal injection. Survival rates of mice were recorded for 7 days.

### Neutropenic mouse thigh infection model

Female BALB/c mice (n = 8 per group) were rendered neutropenic by two consecutive doses of cyclophosphamide (150 and 100 mg/kg delivered on 4 and 1 days before infection). *E. coli* B2 suspension (100 μL, 1.0 × 10^5^ CFUs per mouse) was injected into the right thighs of mice. At one-hour post-infection, mice were intraperitoneally injected with PBS, colistin (10 mg/kg), melatonin (50 mg/kg), or combinations of the two agents (5 + 50 mg/kg, 10 + 20 mg/kg or 10 + 50 mg/kg). At 48 h post-infection, mice were euthanized by cervical dislocation. The right thigh muscle was aseptically removed, homogenized, serially diluted and plated on MHA for CFUs titres.

### Statistical analyses

Statistical analysis was performed using GraphPad Prism 7 and SPSS software. All data are presented as mean ± SD. For the *in vitro* studies, unpaired *t*-test (normally distributed data) between two groups or one-way ANOVA among multiple groups were used to calculate *P*-values. For the *in vivo* studies, n represents the number of animals per group and statistical significance was determined by log-rank (Mantel-Cox) test or the Mann-Whitney U test. Differences with *P* < 0.05 were considered significant. Significance levels are indicated by numbers of asterisks: *P < 0.05, **P < 0.01, ***P < 0.001.

## Results

### Synergistic activity of melatonin with antibiotics

**Figure 1:**
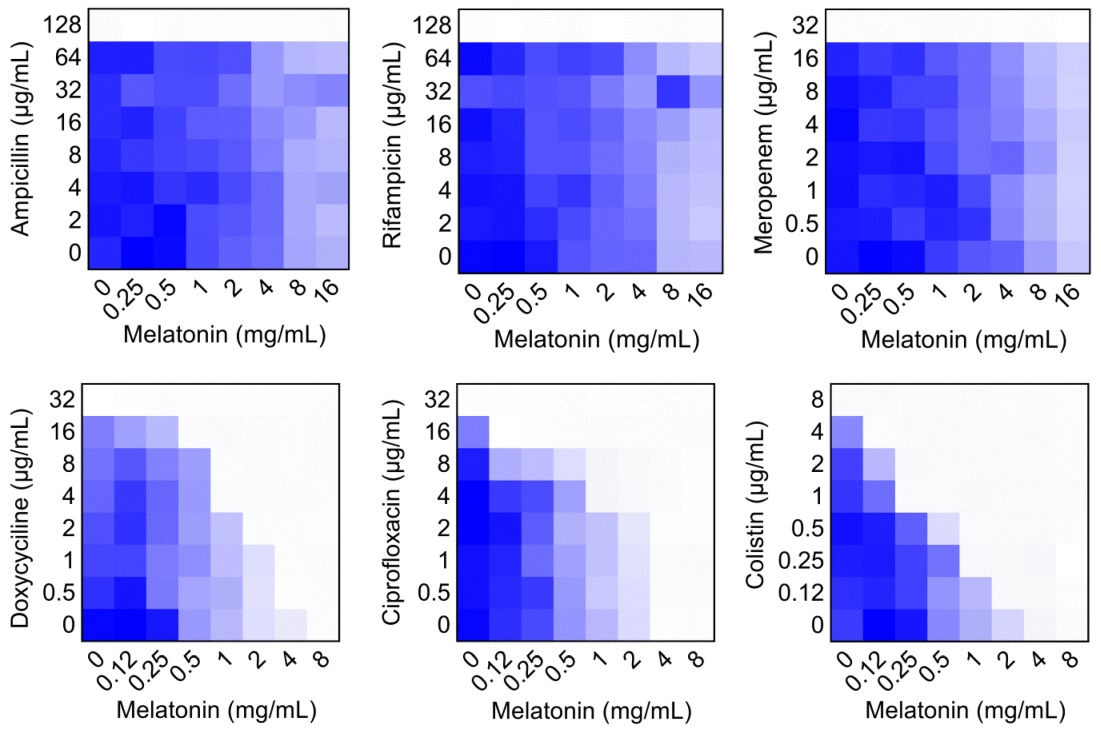
** Checkerboard broth microdilution assays between different classes of antibiotics and melatonin against MCR-1 positive *E. coli* B2, related to Table S1.** Dark blue regions represent higher bacterial cell density. The mean OD at 600 nm of two biological replicates is shown.

To evaluate the potential efficacy of melatonin, checkerboard dilution assays between melatonin and six classes of antibiotics were performed, including ampicillin (target cell wall), rifampicin (RNA synthesis), meropenem (cell wall), doxycycline (protein synthesis), ciprofloxacin (DNA synthesis) and colistin (membrane damage). Interestingly, we found that melatonin potentiated doxycycline, ciprofloxacin and colistin activities against *mcr-1* carrying *E. coli* B2, but not other three antibiotics **(Figure 1)**. Notably, melatonin displayed the highest synergistic activity with colistin (FICI = 0.063), accompanied by a 32-fold decrease in MIC values from 8 μg/mL to 0.25 μg/mL, which is below the clinical breakpoint (2 μg/mL, according to EUCAST 2017 and CLSI 2016) **(Table S2)**. We next tested this synergistic effect in other *mcr* variants or notorious Gram-negative pathogens. As expected, we observed a significant synergy in another bacterium and *mcr-3/mcr-8* carrying Enterobacteriaceae, suggesting a robust potentiation of melatonin with colistin against *mcr*-carrying Gram-negative pathogens **(Figure S1 and Table S3)**. In addition, this synergistic activity was also evidenced in thirteen clinical colistin-resistant *E. coli* from elk** (Figure S2)**.

**Figure 2:**
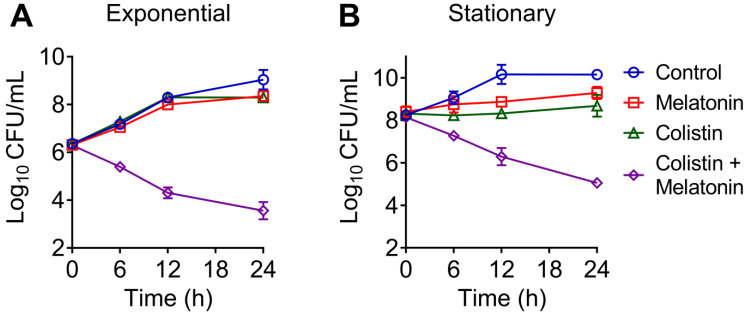
** Time-dependent killing curve of *E. coli* (*mcr-1*) by the combination of melatonin and colistin.** *E. coli* B2 were grown to exponential (**A**) and stationary (**B**) phase, and challenged with melatonin (1 mg/mL) and colistin (2 µg/mL) alone or their combination for 24 h. Data are representative of three independent experiments and shown as mean ± SD.

To further investigate their synergistic bactericidal activity, time-dependent killing curves for different growth phases of bacteria, including exponential and stationary *E. coli*, were determined. It has been demonstrated that many severe human and animal infections are caused by quiescent or slow-growing bacteria, which are difficult to treat by traditional regimens 39. We found that neither melatonin nor colistin monotreatment killed exponentially growing or stationary *E. coli* B2. In contrast, the combination led to a reduction of bacterial load approximate by 4-log_10_ in a growth phase-independent manner **(Figure 2A and 2B)**. These results suggested that melatonin indeed drastically enhances colistin bactericidal activity against MCR-positive pathogens. In addition, we found a weak synergistic effect in MCR-negative pathogens such as *E. coli* MG1655 and *E. coli* ATCC 25922 (FICI = 0.375), and *S. enterica* ATCC 13076 (FICI = 0.14)** (Table S3)**, implying that its mechanism is not limited to the inhibition of bacterial resistance.

### Safety and stability evaluation of colistin and melatonin combination

A critical factor that restricts the colistin application in the clinic is the potential toxicity effects including nephrotoxicity and neurotoxicity 40. To assess whether melatonin influences the toxicity of colistin, we analyzed hemolysis and cytotoxicity of colistin in the presence of melatonin. Encouragingly, we did not observe enhanced toxicity in the combination treatment. Instead we found that melatonin decreased the hemolytic activity of colistin at 128 μg/mL to RBCs by approximately 20% **(Figure S3A)**, and slightly reduced the cytotoxicity of colistin in CHO and HEK293T cells **(Figure S3B)**. We next evaluated the stability of this combination in the presence of serum, DMEM or different salt ions. It has been suggested that divalent cations (Mg^2+^ and Ca^2+^) are essential to bridge negative-charged phosphate groups between the LPS molecules, which helps to avoid the accumulation of repulsive forces and maintain the stability of the bacterial outer membrane 41. Melatonin retained its synergistic activity with colistin in the presence of 10% serum or DMEM **(Table S2)**. In agreement with the assumed effects of divalent cations, we found that EDTA enhanced their synergistic activity against both *E. coli* B2 (*mcr-1*) and *E. coli* ATCC 25922, whereas Mg^2+^ and Ca^2+^ suppressed it, suggesting that the synergistic mechanisms of melatonin may be relevant to the disruption of the bacterial outer membrane **(Figure S4 and Table S2)**. These data also imply that the joint use of colistin and melatonin is safe and stable.

### Structure-activity relationship of melatonin

To gain further insights into the specific moieties of melatonin in enhancing colistin activity against resistant pathogens, we performed structure-activity relationship studies with melatonin and structurally similar molecules **(Table 1)**. Specifically, we found that the indole moiety alone **(7)** has some synergistic activity, and the replacement of the indolic structure by an imidazole ring such as in histamine **(8)** drastically abolished any colistin potentiation, indicating that indole moiety is the basic chemical structure for synergistic activity of melatonin. In addition, the substitution of the ethyl-acetamido residue by 2-aminopropionic acid such as in 5-hydroxy-*L*-tryptophan **(3)** and *L*-tryptophan **(4)** strongly diminished the potentiation toward colistin. Deletion of the *N*-acetyl group as in 5-methoxytryptamine **(6)** retained its activity to colistin. Moreover, serotonin **(1)**, tryptamine **(2)** and *N*-acetyl-5-hydrotryptamine **(5)** displayed synergistic activities similar to that of melatonin, indicating that a 5-methoxy group at ring atom 5 is not required for a synergistic effect on colistin. Consistent with this observation, several tryptamine derivatives were confirmed to sensitize colistin-resistant bacteria to colistin killing 42, 43.

### Melatonin enhances the membrane-damaging ability of colistin

**Figure 3:**
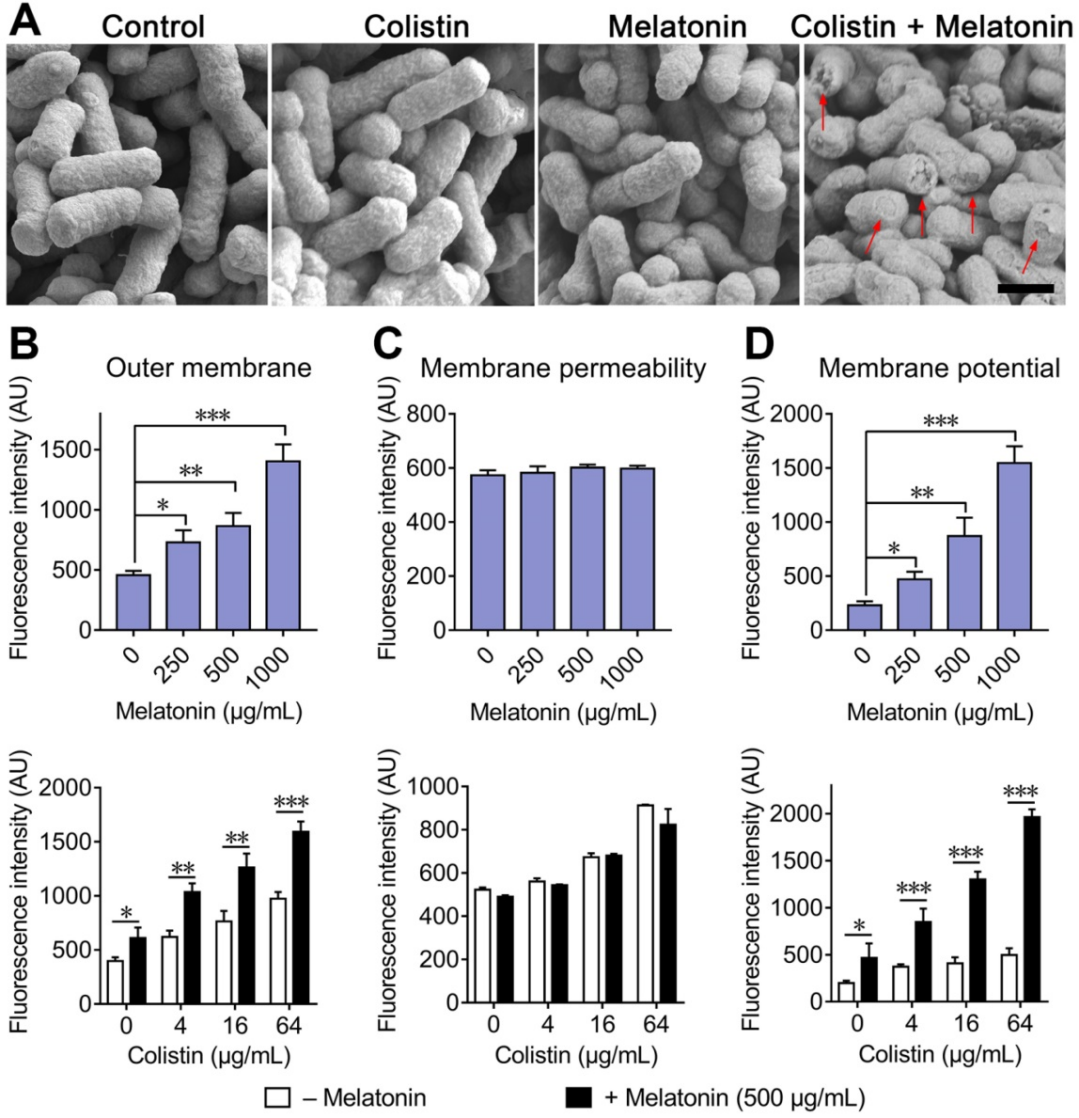
** Melatonin potentiates the damage of colistin to bacterial membrane.** (**A**) Morphological changes of *E. coli* B2 treated with sub-MIC of colistin or melatonin or their combination visualized with SEM. Scar bar, 0.5 µm. Destroyed outer membrane was marked by red arrows. (**B**) Melatonin permeabilizes the outer membrane, and enhances outer membrane disruption of colistin. Permeability was evaluated by measuring the fluorescence intensity of 1-*N*-phenylnaphthylamine (NPN) after 1 h exposure to either increasing concentrations of melatonin, colistin or colistin plus melatonin (500 µg/mL). (**C**) No effect on membrane permeability for propidium iodide (PI) in *E. coli* after treatment with melatonin. (**D**) Melatonin causes dissipation of membrane potential and drastically enhances colistin effects on membrane potential. Fluorescence dye DiSC_3_(5) was used to assess membrane potential changes induced by melatonin, colistin or combination. All experiments were performed with biological replicates and presented as mean ± SD. Unpaired *t*-test between two groups or one-way ANOVA among multiple groups were used to calculate *P*-values (*P < 0.05, **P < 0.01, ***P < 0.001).

Colistin exhibits bactericidal activity against Gram-negative bacteria through specifically interacting with LPS in the bacterial outer membrane. Accordingly, colistin resistance is primarily related to modified LPS and decreased affinity between colistin and components of the bacterial outer membrane. Thus, we first speculated as to whether the addition of melatonin restores colistin ability on disruption of bacterial membrane. To validate our hypothesis, we investigated the morphological changes of *E. coli* treated by sub-MIC of colistin or melatonin and their combination by SEM analysis. Compared with the monotreatment, we observed a significant damage of the outer membrane in the combination group** (Figure 3A)**. To further confirm this, we investigated the outer membrane permeability by means of 1-*N*-phenylnaphthylamine (NPN)** (Figure 3B)**, membrane permeability by propidium iodide (PI) **(Figure 3C)** and the membrane potential using 3,3-dipropylthiadicarbocyanine iodide (DiSC_3_(5))** (Figure 3D)** in *E. coli* B2 (*mcr-1*). Consistently, we found that the addition of melatonin significantly increased outer membrane permeability and caused dissipation of the cytoplasmic membrane potential, but had no effect on whole membrane permeability, indicating that the structural integrity of the inner membrane was largely maintained, although its functionality was affected. Taken together, these results demonstrated that melatonin potentiates colistin activity through enhancing the membrane-damaging ability of colistin.

### Combination of colistin and melatonin promotes oxidative damage, prevents LPS modification and inactivates efflux pump

**Figure 4:**
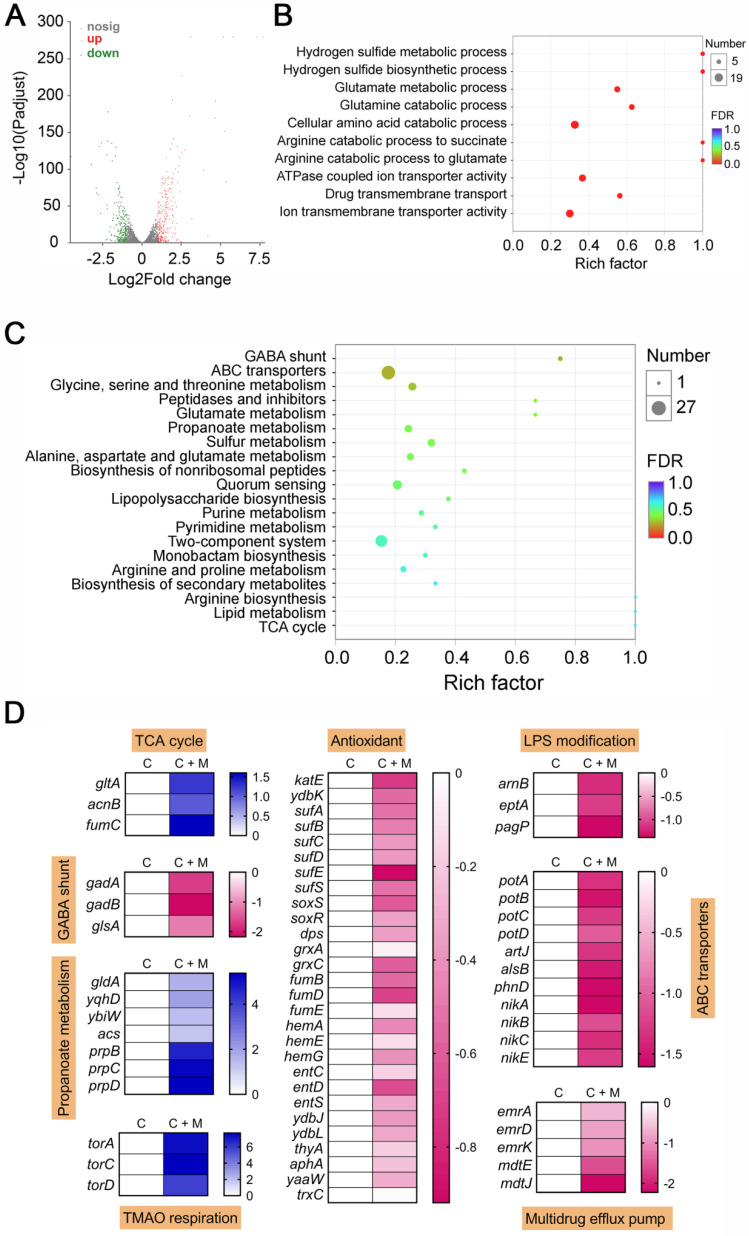
** Transcriptomic analysis of *E. coli* B2 treated by colistin or the combination of colistin plus melatonin.** Volcano plot (**A**), GO (**B**) and KEGG enrichment analysis (**C**) of the differential expression genes (DEGs) in *E. coli* B2 after exposure to colistin or the combination of colistin plus melatonin. The x and y axis in A represent the expression changes and corresponding statistically significant degree, respectively. (**D**) Selected DEGs involved in TCA cycle, GABA shunt, propanoate metabolism, TMAO respiration, antioxidant response, LPS modification, ABC transporters and multidrug efflux pump. C, colistin alone; C + M, the combination of colistin and melatonin.

**Figure 5:**
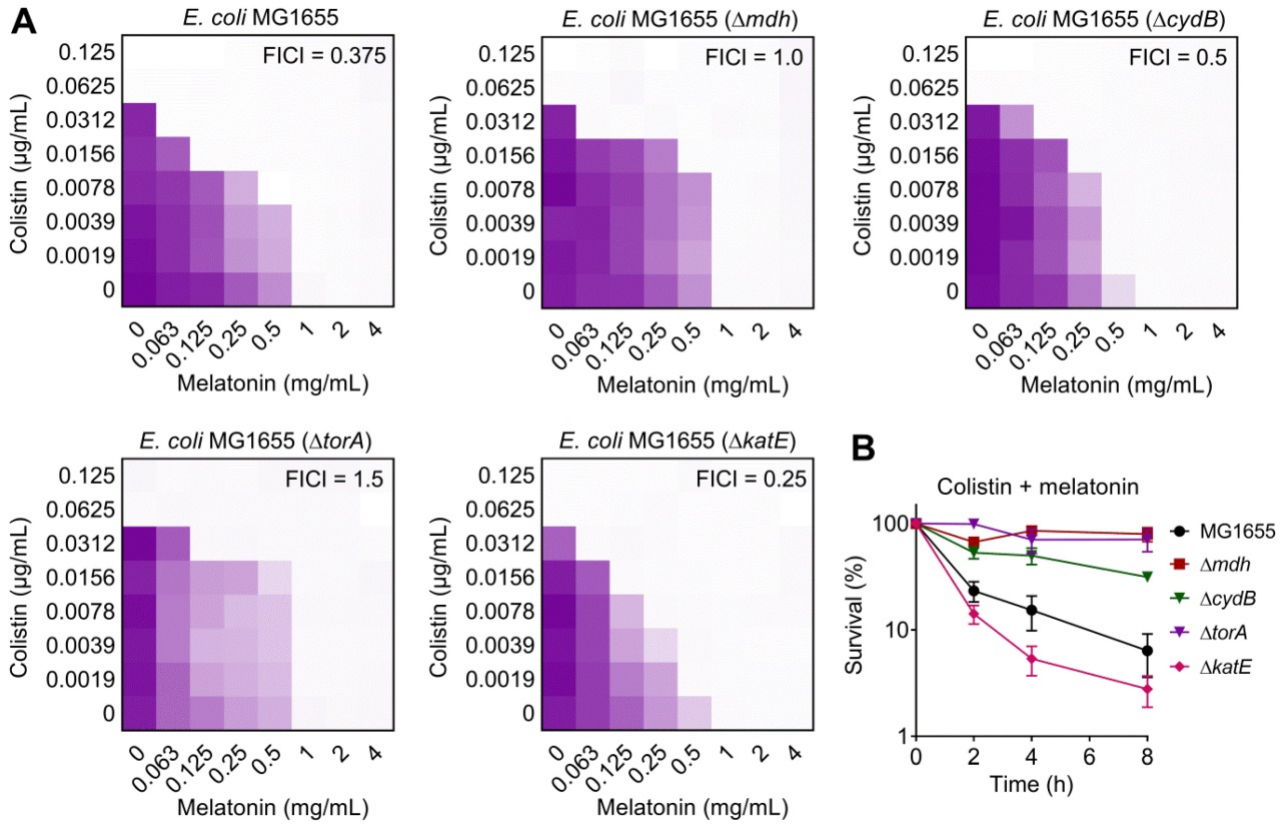
** Effect of deficiency in TCA cycle, electron transport chain, TMAO respiration and antioxidant on synergistic activity of melatonin and colistin against *E. coli*.** (**A**) Checkerboard broth microdilution assays between melatonin and colistin against *E. coli* MG1655 and its gene knockout mutants (Δ*mdh*, Δ*cydB*, Δ*torA* and Δ*katE*), related to Table S3. The mean OD at 600 nm of two biological replicates is presented. (**B**) Survival of *E. coli* MG1655 and its gene knockout mutants (Δ*mdh*, Δ*cydB*, Δ*torA* and Δ*katE*) after the combination treatment of melatonin (1 mg/mL) and colistin (0.0625 µg/mL).

After having demonstrated that melatonin enhances membrane disruption by colistin, the clarification of specific molecular mechanisms is still required. Moreover, the reasons for their weak synergistic activity in *mcr*-negative pathogens remained unclear. To address these issues, we performed transcription analyses of *E. coli* (*mcr-1*) under treatment with colistin or colistin plus melatonin for 4 h. The comparison of treatment with combination to colistin alone revealed an up-regulation of 266 genes and down-regulation of 217 genes (>two-fold) **(Figure 4A)**. Go and KEGG enrichment analysis showed that these differentially expressed genes (DEGs) were involved in GABA shunt, ABC transporters, two-component system, and bacterial metabolism related pathways **(Figure 4B and 4C)**. Specifically, we found that the genes with increased expression were involved in TCA cycle, propanoate metabolism and TMAO respiration, and repressed gene expression in GABA shunt, antioxidant function, LPS modification, ABC transporters and multidrug efflux pumps **(Figure 4D)**. Notably, these multidrug efflux pumps encoded by *emr* or *mdt* genes correlate with colistin resistance in Gram-negative bacteria 44. Expression profiling of representative genes by RT-PCR analysis was consistent with the transcription results **(Figure S5)**. To verify the transcriptome results, we performed gene knockout experiments on related pathways using a reference strain *E. coli* MG1655 that is easily genetically manipulated. TCA cycle knockout strain (Δ*mdh*), electron transport chain (ETC) knockout strain (Δ*cydB*), TMAO respiration knockout strain (Δ*torA*) and antioxidant knockout strain (Δ*katE*) were constructed through homologous recombination. Then, checkerboard broth microdilution assays **(Figure 5A)** and killing curves **(Figure 5B)** were performed. As a result, impaired synergistic activity of melatonin and colistin (FICI ≥ 0.5) were observed on Δ*mdh*, Δ*cydB* and Δ*torA* compared with wild type *E. coli* MG1655. However, Δ*katE* was more sensitive to the combination treatment than wild type. These results suggested that TCA cycle, ETC, TMAO respiration and antioxidant in* E. coli* play a role in the synergistic activity of melatonin and colistin.

**Figure 6:**
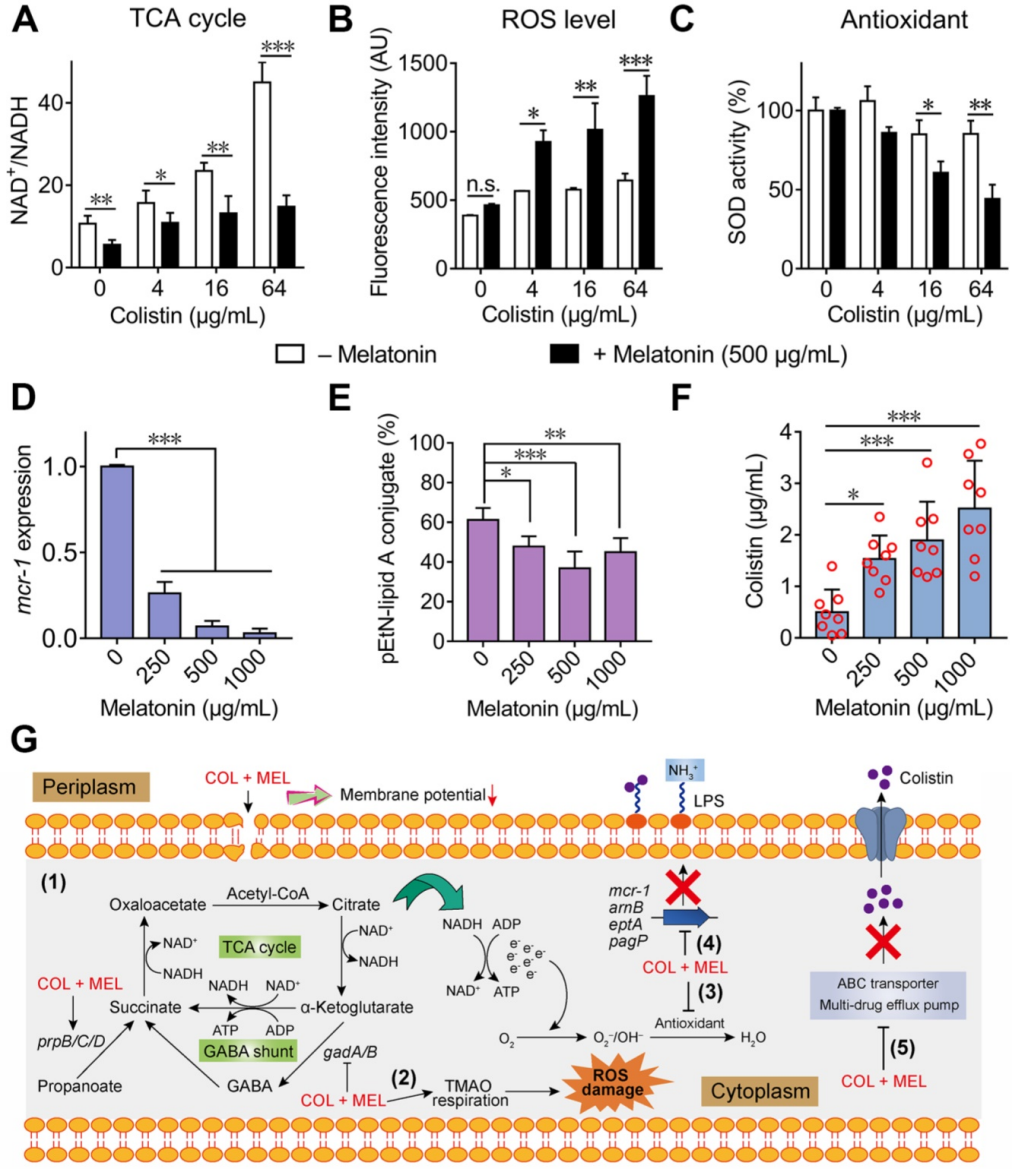
** Synergistic molecular mechanisms of melatonin with colistin against *E. coli*.** (**A**) Accelerated TCA cycle was observed under combination treatment of colistin and melatonin. (**B**) Melatonin supplementation significantly increases the production of ROS level induced by colistin, whereas it alone does not affect ROS levels. *E. coli* B2 was probed by 2′,7′-dichlorodihydrofluorescein diacetate (DCFH-DA) and exposed to colistin in the absence or presence of melatonin (500 µg/mL). After 1 h incubation, the fluorescence of DCF was measured. (**C**) Melatonin supplementation impairs the bacterial oxidative defenses when in combination with colistin. SOD activity in cells was measured by biochemical assay. (**D and E**) Melatonin inhibits expression of resistance gene* mcr-1* (D) and thereby decreases the modification of lipid A by phosphoethanolamine (PE) through MCR-1 (E). The percent of pEtN-lipid A conjugate was determined based on LC-MS/MS analysis. (**F**) Melatonin enhances colistin intracellular accumulation, measured by LC-MS/MS analysis. (**G**) Scheme summarizing the synergistic mechanisms of colistin and melatonin. Combination treatment of colistin and melatonin enhances membrane damage and oxidative damage, inhibits the modification of lipid A and multidrug efflux pump. *P* values (*P < 0.05, **P < 0.01, ***P < 0.001) in (**A-C**) and (**D-F**) were determined by unpaired* t*-test between two groups or one-way ANOVA among multiple groups, respectively. All data are presented as mean ± SD.

To further validate whether the colistin and melatonin combination accelerates the TCA cycle compared with colistin alone, the NAD^+^/NADH ratio in *E. coli* B2 was determined. Consistently, we found that the addition of melatonin significantly decreased the NAD^+^/NADH ratio, indicating an enhanced TCA cycle under combination treatment **(Figure 6A)**. In bacteria, an accelerated TCA cycle is always accompanied by enhanced bacterial respiration and generation of ROS 45. In addition, the transcription analysis revealed that multiple pathways were involved in oxidant damage of *E. coli*. Therefore, we hypothesized that the combination of melatonin and colistin may result in enhanced oxidative damage. To that end, we first determined the generation of ROS and SOD activity by colistin in the absence and presence of sub-MIC of melatonin (500 μg/mL). Consequently, we found that the combination of colistin and melatonin drastically promoted the generation of total ROS **(Figure 6B)** and decreased SOD activity compared with colistin alone **(Figure 6C)**. However, melatonin alone had no direct effect on the total ROS level and SOD activity **(Figure S6A and S6B)**. In cells, ROS include superoxide (O_2_^•-^), hydrogen peroxide (H_2_O_2_) and hydroxyl radical (OH^•^). Interestingly, we also found that melatonin significantly promoted production of H_2_O_2_ in a dose-dependent manner, in both single or combination treatments **(Figure S6C and S6D)**. Additionally, melatonin resulted in enhanced TMAO respiration, which also correlates with the generation of ROS 46. Taken together, we conclude that the combination of colistin and melatonin leads to increased ROS damage through promoting TCA cycle and TMAO respiration, and inhibiting the bacterial antioxidant system. Consistently, addition of ROS scavengers including *N*-acetylcysteine (NAC) 47 and thiourea 48 partially abolished the potentiation of melatonin to colistin **(Table S2)**, indicating that ROS are involved in their synergistic activity.

Considering that the addition of melatonin inhibited the LPS modification related gene expression, as revealed by RNA sequencing **(Figure 4D)**, we determined the *mcr-1* expression upon treatment with melatonin with or without colistin. As expected, the *mcr-1* expression in *E. coli* B2 was down-regulated in the presence of melatonin **(Figure 6D)**. To investigate whether this inhibition could eventually prevent the LPS modification by phosphoethanolamine (PE), the proportion of modified-LPS in *E. coli* (*mcr-1*) under different concentrations of melatonin was measured by LC-MS/MS. Consistently, we observed a decreased pEtN-lipid A conjugate (1920.3 Da) in *E. coli* after melatonin treatment **(Figure 6E)**. Since supplementation with melatonin down-regulated the ABC transporter and multidrug efflux pump, we hypothesized that melatonin may enhance intracellular colistin accumulation. To test this, we evaluated colistin concentrations in *E. coli* upon co-incubation with varying doses of melatonin. We found that melatonin indeed enhanced colistin in cells in a dose-dependent manner **(Figure 6F)**. Collectively, these data demonstrated that melatonin enhances colistin activity by promoting oxidative damage, preventing LPS modification and efflux pump function **(Figure 6G)**.

### Melatonin restores colistin activity* in vivo*

**Figure 7:**
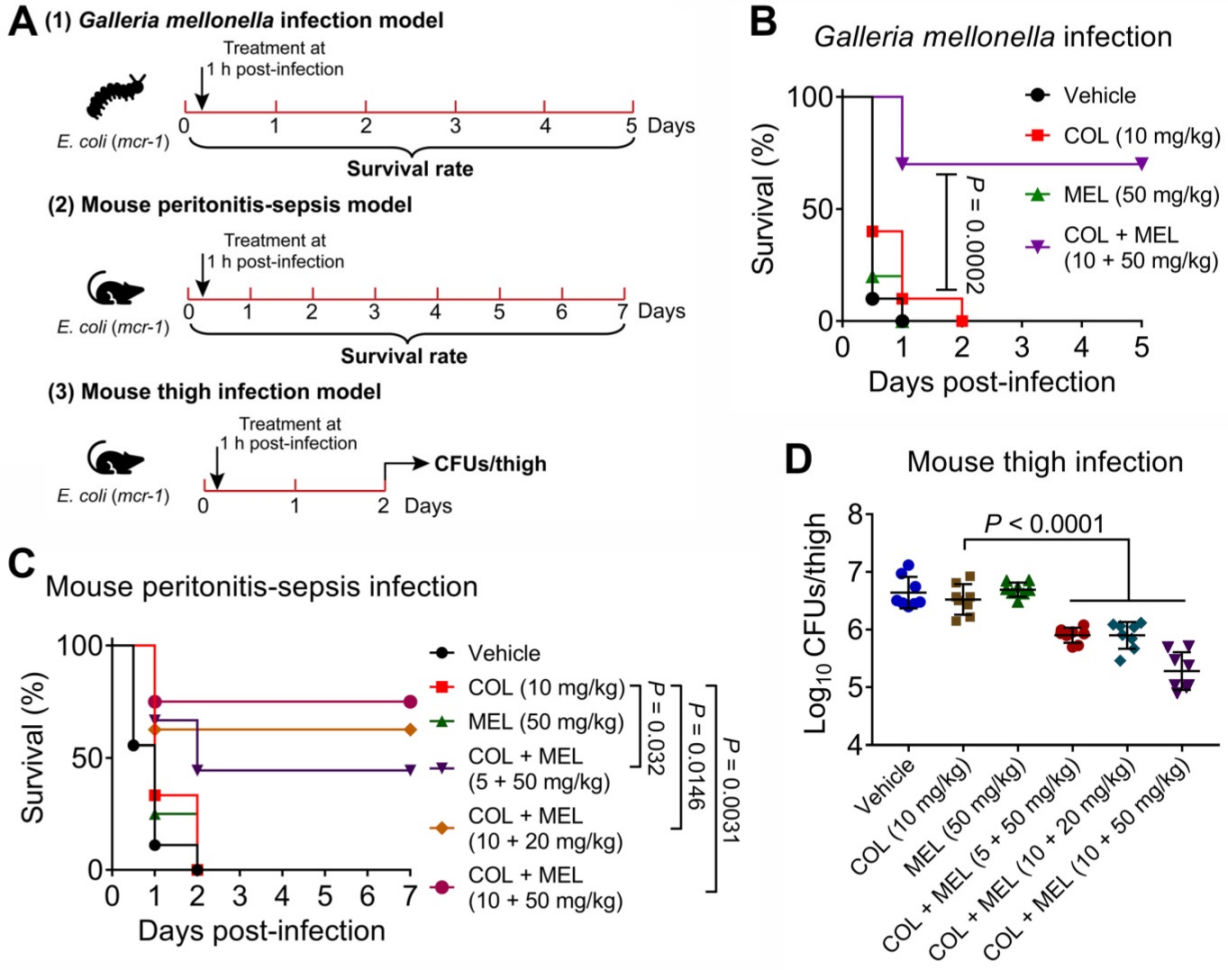
** Melatonin rescues colistin activity in three animal infection models.** (**A**) Scheme of the experimental protocols for three animal infection models. In three infection models, animals were infected by colistin-resistant *E. coli* B2 (*mcr-1*), and treated with a single dose of colistin, melatonin or their combination at one-hour post-infection. In the *Galleria mellonella* infection model (1), survival rate of larvae (n = 10 per group) was monitored during 5 days, related to Figure 7B. In the mouse peritonitis-sepsis infection model (2), the percentage of surviving mice (n = 8 or 9 per group) was recorded during 7 days, related to Figure 7C. In neutropenic mouse thigh infection model (3), thigh muscle bacterial loads in single and combination therapy (n = 8 per group) were counted at 2 days post-infection, related to Figure 7D. (**B**) Combination of colistin (10 mg/kg) and melatonin (50 mg/kg) significantly improved survival rate of *G. mellonella* larvae (n = 10 per group) infected by colistin-resistant *E. coli* (*mcr-1*) compared with colistin monotherapy (10 mg/kg). *P* values were determined by log-rank (Mantel-Cox) test. (**C**) Combination of colistin and melatonin profoundly increased survival rate of mice infected by colistin-resistant *E. coli* (*mcr-1*) during 7 days post-infection in a dose-dependent manner. BALB/c mice (n = 8 or 9 per group) were given a lethal dose of *E. coli* (3.0 × 10^8^ CFUs), and treated with a single dose of colistin (10 mg/kg), melatonin (50 mg/kg), a combination of colistin plus melatonin (5 + 50 mg/kg, 10 + 20 mg/kg and 10 + 50 mg/kg), or PBS by intraperitoneal injection. *P* values were determined by log-rank (Mantel-Cox) test. (**D**) Decreased bacterial load in the neutropenic mouse thigh infection model by combination therapy. Neutropenic BALB/c mice (n = 8 per group) were intramuscularly given a non-lethal dose of *E. coli* (1.0 × 10^5^ CFUs), and treated with a single dose of colistin (10 mg/kg), melatonin (50 mg/kg), a combination of colistin plus melatonin (5 + 50 mg/kg, 10 + 20 mg/kg and 10 + 50 mg/kg), or PBS by intraperitoneal injection. *P* values were determined by Mann-Whitney U test. Data are presented as mean ± SD.

Given that the combination of colistin and melatonin displayed excellent synergistic bactericidal activity against active and dormant pathogens *in vitro*, we reasoned that melatonin would reverse MCR-mediated colistin resistance *in vivo* and thereby restore its clinically efficacy. To that end, we first explored their pharmacokinetic characters after a single *i.p.* injection in mice. Consequently, we found that these two drugs exhibited similar serum drug concentration-time curves and pharmacokinetic parameters, e.g., T_max_ and MRT, implying that they could make full use of their synergistic activity *in vivo* **(Figure S7A and 7B)**. Then, we tested *in vivo* efficacy of this combination in three animal infection models **(Figure 7A)**. In a *Galleria mellonella* infection model, insect larvae after infection by *E. coli* B2 (*mcr-1*) with PBS or colistin treatment all died within 48 hours. However, the combination therapy resulted in 70% survival, which was significantly higher than that obtained by the monotherapy (*P* = 0.0002) **(Figure 7B)**. This survival advantage was also validated in a mouse peritonitis-sepsis model using *E. coli* B2 (*mcr-1*). Remarkably, although colistin or melatonin alone did not prevent a lethal infection by MCR-1-positive *E. coli*, a single dose of the combination treatments led to increased survival of mice at 7 days following infection **(Figure 7C)**. Finally, this combination was tested in a neutropenic mouse thigh infection model. Similarly, three combinations of colistin and melatonin significantly reduced the bacterial load in mouse thigh muscle (*P* < 0.0001) compared with colistin monotherapy **(Figure 7D)**. These data confirmed that melatonin dramatically rescues colistin activity *in vivo*.

## Discussion

Infectious diseases caused by Gram-negative bacteria are a matter of global concern due to limited and ineffective treatments in the clinic 49. Despite the notion that colistin has been widely recognized as one of critical clinically relevant antibiotics against Gram-negative bacteria, MCR-mediated acquired colistin resistance severely diminishes its clinical efficacy. Therefore, the identification of potent adjuvants to rescue colistin activity is of great importance. Although multiple biofunctions of melatonin in prevention and treatment of diseases such as cardiac and brain ischemia-reperfusion injury 50, retinal neovascularization and neuroglial dysfunction 51, obesity 52, and breast cancer 53 have been demonstrated, its potential in bacterial diseases has not been fully explored. In this study, despite the weak antibacterial effect of melatonin at low dosage on bacteria 54, we unexpectedly found that melatonin exhibits the highest potentiation (8 to 32-fold) with colistin in resistant bacteria. Additionally, this activity is independent of bacterial species and resistance gene types. To our knowledge, this study is the first to employ the co-application of melatonin and colistin to treat infectious diseases caused by resistant bacteria.

Importantly, we found that the addition of melatonin slightly reduces the *in vitro* toxicity of colistin. An important reason that limits the clinical use of colistin is its neurotoxicity and nephrotoxicity in mammals 40. The discovery of novel detoxification agents for colistin is meaningful. For example, minocycline 55 and rapamycin 56 were found to attenuate colistin-induced neurotoxicity via suppression of oxidative stress and mitochondrial dysfunction. In particular, one study has indicated that melatonin (5 mg/kg) effectively attenuated colistin-induced nephrotoxicity in rats 57. However, the detailed detoxification mechanism of melatonin is still unknown.

In the experiments on their synergistic mechanisms, an intriguing phenotype is the discovery of the restored affinity between colistin and bacterial modified-LPS in the presence of melatonin, which may be an indispensable change that accounts for their synergistic activity. In-depth mechanistic analysis showed that melatonin potentiates colistin activity through multiple pathways, including the promotion of oxidative damage, inhibition of LPS modification by PE and deprivation of multidrug efflux pump functions. Notably, the promotional effect on ROS generation in bacteria appears to be inconsistent with the previous notion that melatonin alone possesses antioxidant activity in normal mammalian cells 58. There are several reasons that may account for this seemingly controversial phenomenon. First, melatonin alone would not stimulate the increase of total ROS level in *E. coli*. Increased ROS levels are only observed in combination treatment of the two drugs. Secondly, the antioxidant activity of melatonin strongly depends on the possible electron transfer reactions with the respective ROS. Thus, melatonin is an efficacious scavenger of OH^•^ 59, and other free radicals that are capable of undergoing single-electron transfer reactions 60, but has less effect on O_2_^•-^ and H_2_O_2_. By contrast, we found that melatonin significantly facilitates the production of H_2_O_2._ Of additional interest is that Aghdam *et al.* also found that melatonin treatment triggers H_2_O_2_ accumulation in strawberry fruits 61. Moreover, melatonin is known to exert prooxidant effects in mammalian tumor cells, in the context of its pro-apoptotic actions on transformed cells 62, 63. In both tumor and nontumor cells, melatonin turned to prooxidant behavior under conditions of apoptosis induction at highly elevated concentrations 64, 65. Collectively, these findings underline melatonin's potential of acting in a prooxidant manner. In the case of Gram-negative bacteria, we speculate that melatonin, when combined with colistin, enhances the antibiotic-induced oxidative damage by accelerating the TCA cycle and TMAO respiration, regardless of the partial clearance of total ROS. This mode of action partially explains why this synergistic activity is also applicable in *mcr*-negative bacteria. In addition, melatonin reduces the expression of LPS modification and multidrug efflux pump associated genes. Consistently, decreased pEtN-lipid A conjugate in *E. coli* (*mcr-1*) by melatonin is observed. This also explains our earlier observation that melatonin restored the membrane-damaging ability of colistin in resistant pathogens. Meanwhile, increased antibiotic accumulation in *E. coli* is found, which are necessary for antibiotic killing of Gram-negative bacteria 66. These findings on molecular mechanisms of melatonin undoubtedly provide a basis allowing screening for other, perhaps more effective antibiotic adjuvants. Nevertheless, in the future work, more in-depth studies on each synergistic pathway of melatonin are still required to provide a better understanding of its modes of action. In addition to these *in vitro* synergistic mechanisms, the beneficial effects of melatonin in immunomodulation 67 and anti-inflammation 68 that have been widely reported in mammals may contribute to improving the *in vivo* efficacy of this combination.

In conclusion, our data have shown that the FDA-approved melatonin exhibits a potent synergistic activity with several antibiotics, in particular, colistin against resistant pathogens both *in vitro* and *in vivo*. The discovery of melatonin as a novel colistin adjuvant highlights the huge potential of non-antibiotic agents against bacterial infectious diseases. We posit that melatonin and, perhaps, analogues thereof represent attractive lead compounds for antibiotic adjuvants to address the increasing threat by infections with colistin-resistant Gram-negative bacteria.

## Supplementary Material

Supplementary figures and tables.

## Figures and Tables

**Table 1:** Structure-activity relationship of melatonin with colistin against *E. coli* (*mcr-1*)

Analogues	Chemical structure	MIC^a^ (μg/mL)	FIC index	MIC^b^ (μg/mL)	Potentiation (fold)^c^
Melatonin	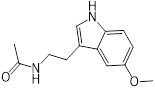	8	0.063	0.25	32
1 (Serotonin)	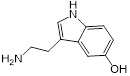	8	0.188	1	8
2 (Tryptamine)	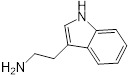	8	0.156	0.25	32
3 (5-Hydroxy-*L*-tryptophan)	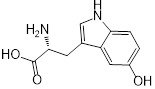	8	1	4	2
4 (*L*-tryptophan)	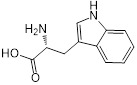	8	2	8	1
5 (*N*-acetyl-5-hydroxytryptamine)	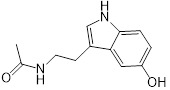	8	0.094	0.25	32
6 (5-Methoxytryptamine)	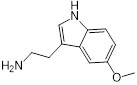	8	0.156	0.25	32
7 (Indole)	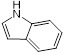	8	0.154	1	8
8 (Histamine)	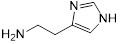	8	>4	>32	-

^a/b^ MICs of colistin in the absence or presence of sub-MIC of melatonin and its analogues; ^c^ Degree of colistin potentiation in the presence of sub-MIC of melatonin and its analogues.

## Abbreviations

MCR: mobilized colistin resistance
FDA: Food and Drug Administration
MBLs: metallo-β-lactamase
PBP2a: penicillin-binding protein 2a
LPS: lipopolysaccharides
MRSA: methicillin-resistant *Staphylococcus aureus*
MHB: Mueller-Hinton Broth
RBCs: red blood cells
CHO: Chinese Hamster Ovary cell
HEK293T: Human Embryonic Kidney cell
DMEM: Dulbecco's modified Eagle's medium
FBS: fetal bovine serum
MIC: minimum inhibitory concentration
FICI: fractional inhibitory concentration index
RT-PCR: real-time reverse transcriptase-polymerase chain reaction
GABA: γ-aminobutyric acid
ROS: reactive oxygen species
SOD: superoxide dismutase
OD: optical density
MIC: minimum inhibitory concentration
COL: colistin
MEL: melatonin
