# Supplementary material for: Melatonin overcomes MCR-mediated colistin resistance in Gram-negative pathogens
Source: Theranostics. 2020 Aug 29;10(23):10697–711. doi: 10.7150/thno.45951 (PMC7482817; doi:10.7150/thno.45951)
Supplement: Supplementary file 1 — Supplementary figures and tables. [file thnov10p10697s1.pdf]

Supplementary Material

Figures

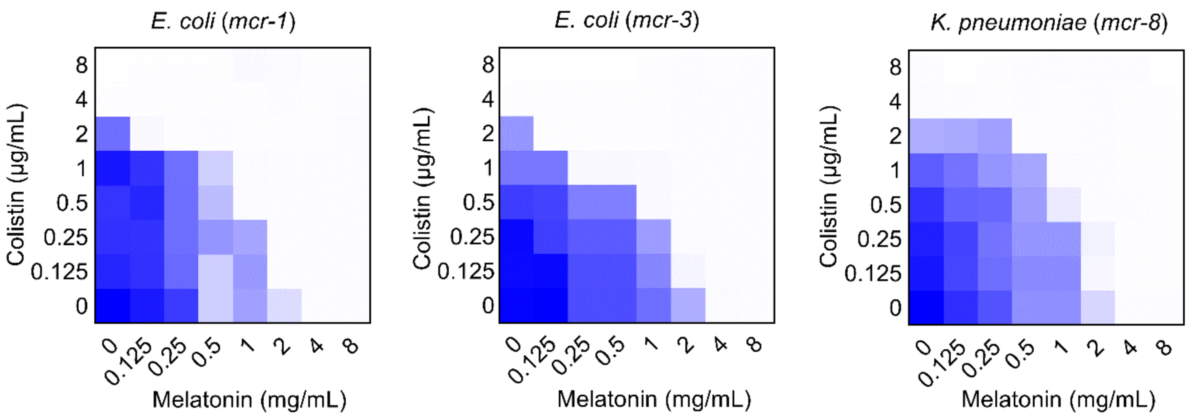

Figure S1 Melatonin potentiates colistin activity against both *mcr*-carrying and colistin-resistant Gram-negative pathogens, related to Table S3.

Dark blue regions represent higher cell density. Data represent the mean OD (600 nm) of two biological replicates.

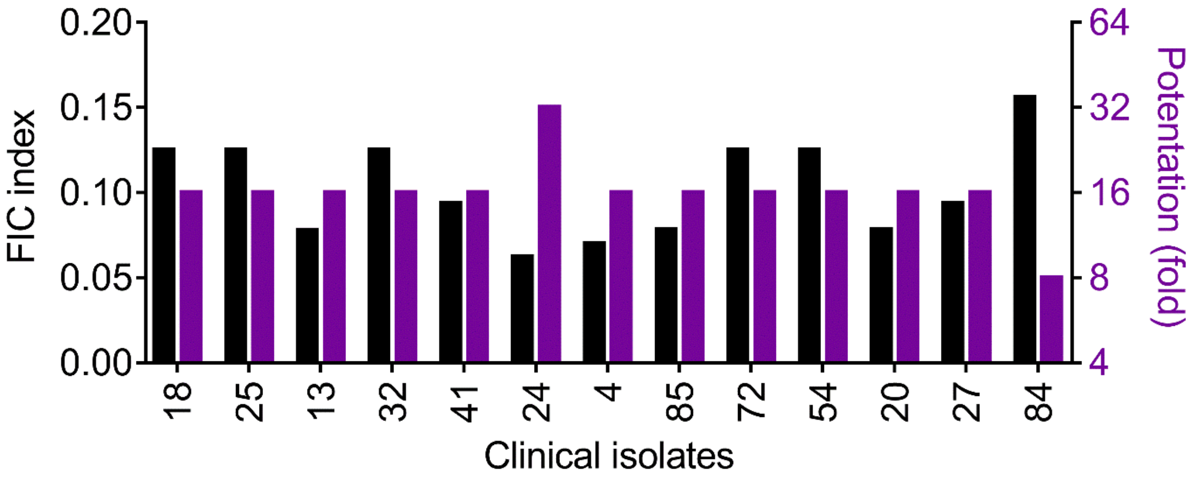

Figure S2 Synergistic activity between melatonin and colistin against clinical colistin-resistant isolates from elk.

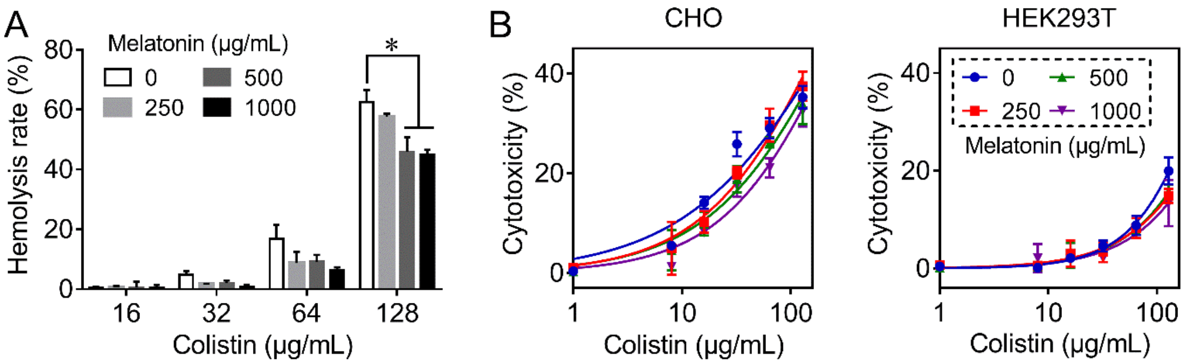

Figure S3 Melatonin moderately alleviates the hemolysis to RBCs (A) and cytotoxicity in CHO and HEK293T cells (B) caused by colistin.

Data are presented as mean ± SD and differences in (A) were assessed by one-way ANOVA and denoted as follows: *$P < 0.05$.

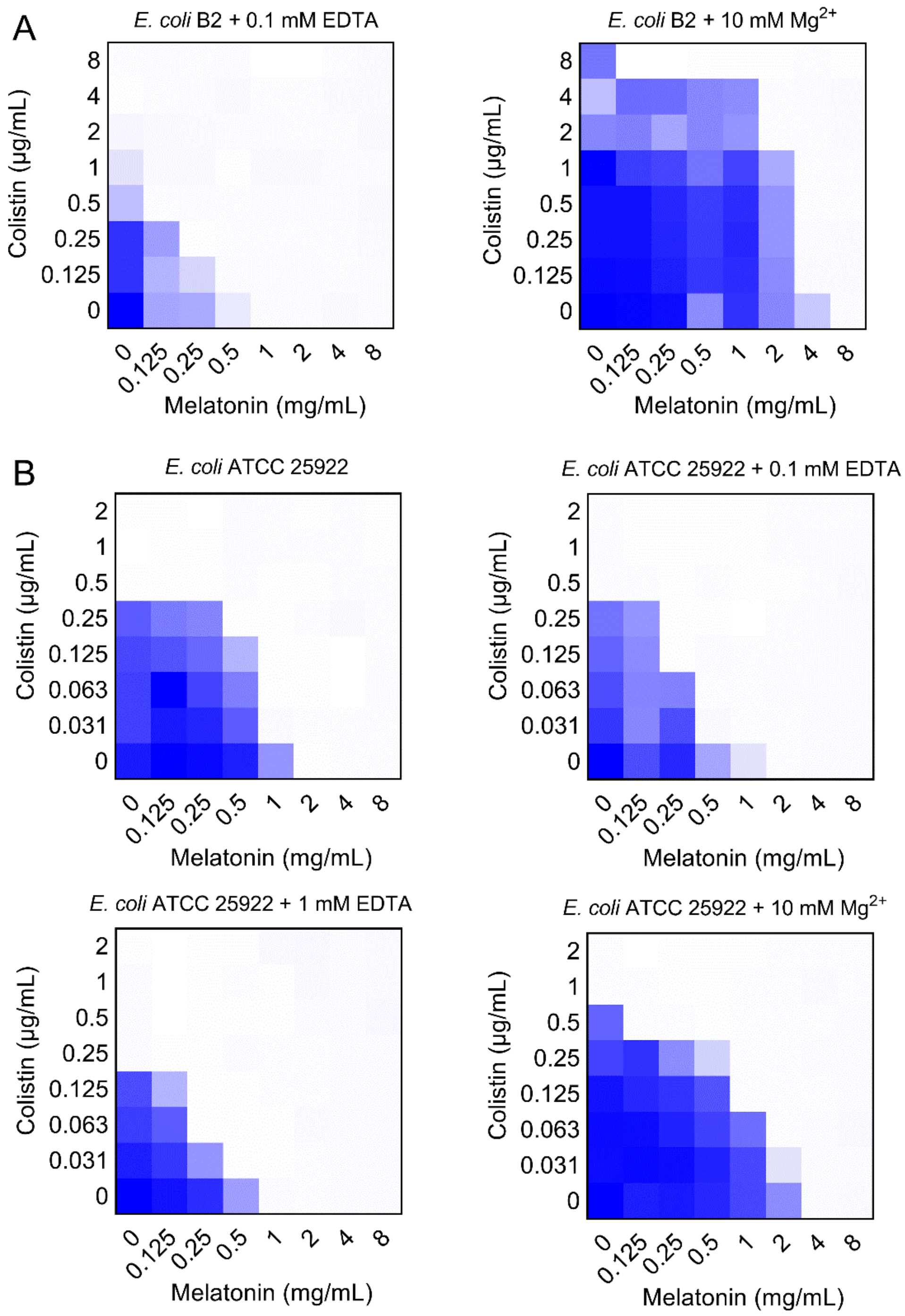

Figure S4 Effect of EDTA or Mg^{2+} on the synergistic activity between melatonin and colistin against *E. coli* B2 (A) and *E. coli* ATCC 25922 (B), related to Table S2.

Dark blue regions represent higher cell density. Data represent the mean OD (600 nm) of two biological replicates.

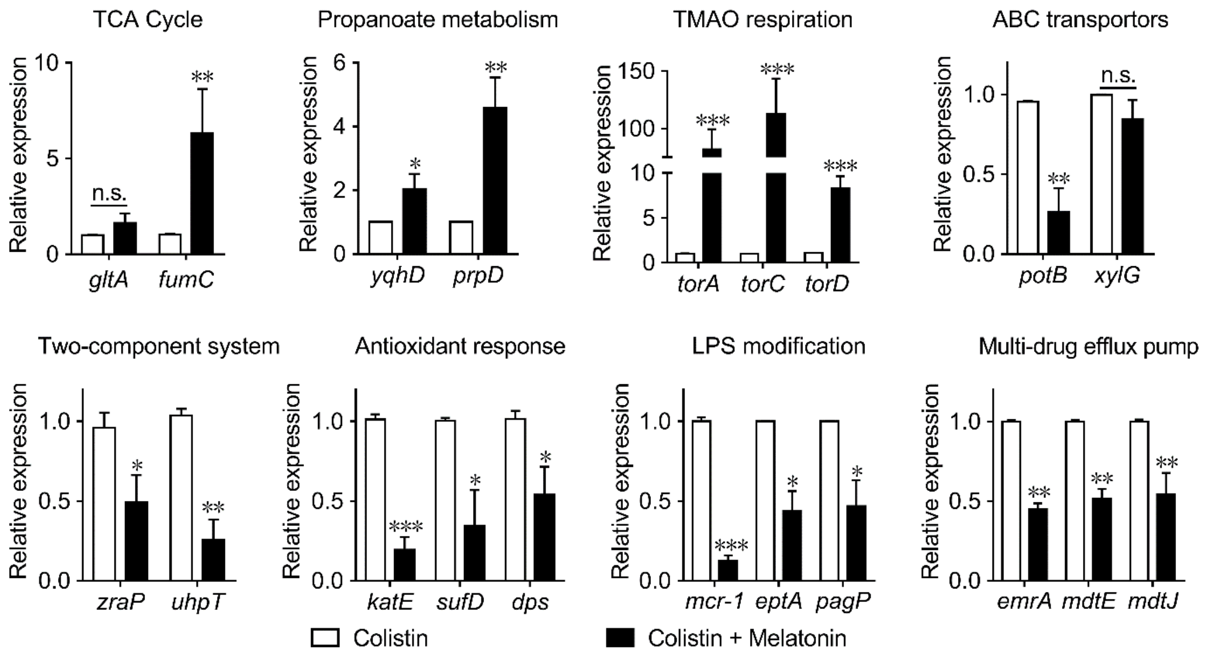

Figure S5 Relative expression of representative genes of *E. coli* B2 after treatment with colistin (40 μg/mL) alone or in combination of melatonin (1 mg/mL) for 4 h by RT-PCR. Data are presented as mean ± SD and unpaired *t*-test was used to calculate *P*-values (**P* < 0.05, ***P* < 0.01, ****P* < 0.001). n.s., not significant.

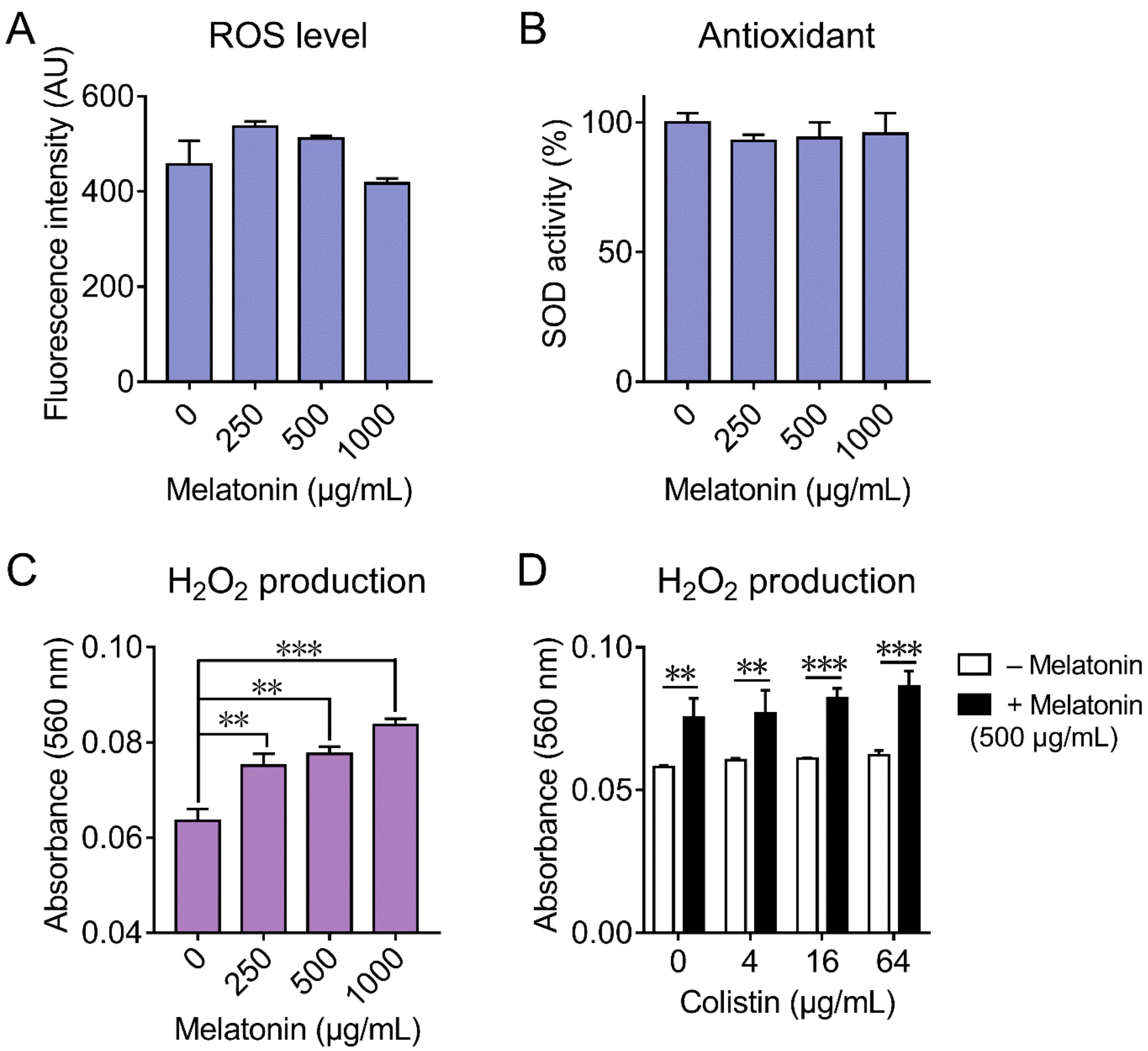

Figure S6 Effect of melatonin on ROS production and SOD activity in *E. coil*.

(A and B) Melatonin monotreatment has no effect on total ROS level (A) and SOD activity (B). **(C and D)** Melatonin induces the production of H_2O_2 in the absence (C) and presence of colistin (D). *$P < 0.05$, **$P < 0.01$, ***$P < 0.001$, determined by one-way ANOVA for multiple groups or unpaired *t*-test for two groups. All data are presented as mean ± SD.

A

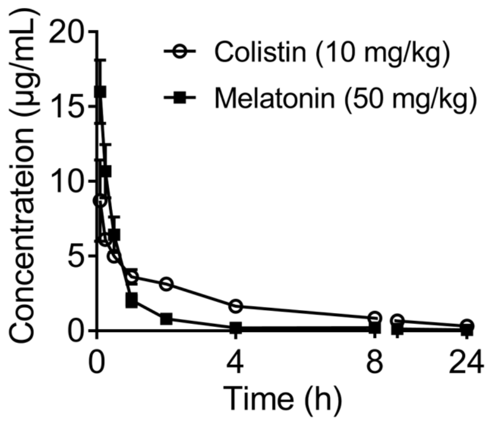

B

Parameters	Colistin	Melatonin
AUC_{last} (h×μg/mL)	26.96±2.76	12.04±2.22
Cmax (μg/mL)	8.71±2.73	15.99±3.66
Tmax (h)	0.083	0.083
Vz_F	3.77±0.61	80.04±77.88
Cl_F	0.37±0.039	4.26±0.86
HL_Lambda_z (h)	7.04±1.30	11.78±9.27
Lambda_z (h)	0.10±0.018	0.08±0.046
MRTINF_obs (h)	8.81±2.41	5.08±2.46

Figure S7 Pharmacokinetic analysis of colistin and melatonin in mice.

(**A**) The mean plasma concentrations of colistin and melatonin after a single *i.p.* injection of 30 mg/kg tigecycline and 10 mg/kg azidothymidine (3 mice per time point). All data are presented as mean ± SD.

(B) Pharmacokinetic parameters of colistin and melatonin calculated with a non-compartmental analysis model based on WinNonlin. AUC_{last}: area under the plasma concentration-time curve from time 0 to 24 h; Cmax: maximal plasma concentration; Tmax: time to maximal plasma concentration; Vz_F: the volume of distribution estimated based on total AUC; Cl_F: total body clearance; HL_Lambda_z: terminal half-life of the drug; Lambda_z: the elimination rate constant estimated from the regression line representing the terminal phase of the concentration-time profile; MRTINF_obs: the mean residence time from the first sampling time extrapolated to infinity based on the last observed concentration.

Tables

Table S1 Strains of bacteria used in this study.

Strains	Source
Escherichia coli	ATCC 25922
E. coli MG1655	ATCC 700926
E. coli MG1655 (Δ*mdh*)	In this study
E. coli MG1655 (Δ*cydB*)	In this study
E. coli MG1655 (Δ*torA*)	In this study
E. coli MG1655 (Δ*katE*)	In this study
E. coli C3 (NDM-1)	In this study
E. coli G6 (NDM-5)	In this study
E. coli B2 (NDM-5 + MCR-1)	In this study
E. coli G92 (MCR-1)	In this study
E. coli CP131 (MCR-3)	In this study
E. coli 18 (MCR-1)	In this study
E. coli 25 (MCR-1)	In this study
E. coli 13 (MCR-1)	In this study
E. coli 32 (MCR-1)	In this study
E. coli 41 (MCR-1)	In this study
E. coli 24 (MCR-1)	In this study
E. coli 4 (MCR-1)	In this study
E. coli 85 (MCR-1)	In this study
E. coli 72 (MCR-1)	In this study
E. coli 54 (MCR-1)	In this study
E. coli 20 (MCR-1)	In this study
E. coli 27 (MCR-1)	In this study
E. coli 84 (MCR-1)	In this study
Klebsiella pneumoniae D120 (MCR-8)	In this study
Salmonella enterica	ATCC 13076

ATCC, American Type Culture Collection.

Table S2 Synergistic activity of melatonin in combination with different classes of antibiotic against *E. coli* B2, related to Figure 1.

Targets	Antibiotics	MIC[a] (μg/mL)	FIC index	MIC[a] (μg/mL)	Potentiation (fold)[c]
Cell wall	Ampicillin	128	2	128	–
RNA synthesis	Rifampicin	128	2	128	–
Cell wall	Meropenem	32	2	32	–
Protein	Doxycycline	32	0.250	4	8
DNA synthesis	Ciprofloxacin	32	0.156	4	8
Cell membrane	Colistin	8	0.063	0.25	32
	+ 10% serum	8	0.094	0.5	16
	+ 10% DMEM	8	0.094	0.5	16
	+ 0.1 mM EDTA	2	0.039	0.0156	128
	+ 10 mM Mg^{2+}	16	0.375	2	8
	+ 10 mM Ca^{2+}	16	0.250	2	8
	+ 10 mM Na^{+}	8	0.094	0.5	16
	+ 10 mM K^{+}	8	0.188	0.5	16
	+ 10 mM NAC	32	0.188	4	8
	+ 10 mM Thiourea	8	0.125	0.5	16

[a/b]MICs of antibiotics in the absence or presence of sub-MIC of melatonin.

[c]Degree of antibiotics potentiation in the presence of sub-MIC of melatonin.

–, none of potentiation activity.

Table S3 Potentiation activity of melatonin with colistin against MCR-negative or positive Gram-negative pathogens, related to Figure S1.

Pathogens and genotypes	MIC[a] (μg/mL)	FIC index	MIC[a] (μg/mL)	Potentiation (fold)[c]
**MCR-negative pathogens**				
E. coli MG1655	0.0625	0.375	0.0156	4
E. coli MG1655 (Δ*mdh*)	0.0625	1.0	0.0312	2
E. coli MG1655 (Δ*cydB*)	0.0625	0.5	0.0156	4
E. coli MG1655 (Δ*torA*)	0.0625	1.5	0.0312	2
E. coli MG1655 (Δ*katE*)	0.0625	0.25	0.0078	8
E. coli ATCC 25922	0.5	0.375	0.125	4
E. coli C3	0.125	0.266	0.0312	4
E. coli G6	0.125	0.375	0.0156	8
S. enterica ATCC 13076	0.125	0.14	0.0156	8
**MCR-positive pathogens**				
E. coli G92 (*mcr-1*)	4	0.156	0.5	8
E. coli CP131 (*mcr-3*)	4	0.156	0.5	8
K. pneumoniae D120 (*mcr-8*)	4	0.25	0.5	8

[a/b] MICs of antibiotics in the absence or presence of sub-MIC of melatonin.

[c] Degree of antibiotics potentiation in the presence of sub-MIC of melatonin.

Table S4 RT-PCR primers used in this study.

Genes	Sequence (5'→3')	Product (bp)
gltA	CACCCTCAACGGGGATACAG	132
	GATGCGGTTGAAGTGAAGCC	
fumC	TACTTCGCTCACGACATCCG	118
	CCTGACGGTTGATCTTCGCT	
yqhD	GCGTAGTGGACGCCTTTGTA	127
	TTCAGGGCTTTCGGACCATC	
prpD	GCAGTTGAAGCAGCGATGAC	89
	TGGGTGCGAATGGTCACTTT	
torA	GCTGAAAAACCAGCAAGCGA	73
	CGCTTTTAGCTGCGCGTAAT	
torC	TGGGCTTATGCGGATTCGTT	79
	TAGCGTCAAAGTGGGCGATT	
torD	AGAGTTTGTTGCGCGTTGTC	88
	GTCGCACTCCACTAACACCA	
potB	TTGGCACCAGCTTTTTGACC	130
	ATCAGCGCCATATTCAGCGA	
xylG	CCAAAACCTTCGGCAGTGTG	71
	TGAGACGATTTCGCCAGCAT	
zraP	ACGGAACACGAAAATTGCCC	128
	GTTGTTCGCTGGTCAAAGGC	
uhpT	TGGGCTTCAGTGCCAGTATG	129
	GCGTCCATTTGGTGATGGTG	
katE	CCGGAATACGAACTGGGCTT	125
	ATTTTGCCGACACGCTGAAC	
sufD	TCAATGGCGAAAACAGCACG	127
	GTTTTGTGCAACTGTCGGCT	
dps	ATCAACAGCAAAACCCCGCT	145
	CTGCGGTGTCGTCATCTTTC	
mcr-1	AAAGACGCGGTACAAGCAAC	213
	GCTGAACATACACGGCACAG	
eptA	TTACAACCGCTATCCGCCTC	111
	ACCAGCGTGTTGTCGTAAGT	
pagP	GTAACGGCACGCGATAACTG	70
	CATAACCCACGGAGGCCAAT	
emrA	CCAAAACTGCACTGGCTTCC	125
	GCGGTTGTAGTCGCTTTGTG	
mdtE	TTACGGGCGATTTTCCCCAA	88
	CATTCTGGCGGCTACCTTCA	
mdtJ	CTATTACGCTCTGCCCTCGG	117
	GGGGACAAATCCGCCAAGTA	

Table S5 MRM parameters for the determination of colistin and melatonin by LC-MS/MS.

Compounds	Precursor ions (*m/z*)	Daughter ions (*m/z*)	DP (V)	CE (eV)
Colistin	390.8	385.1	32	15.00
		101.1*	32	23.86
Melatonin	233	143.0	40	38.99
		131.0*	40	48.99

*, quantitative ion; DP, declustering potential; CE, collision energy.

Table S6 Regression equation, LOD and LOQ of colistin and melatonin.

Compounds	Regression equation	R^2	LOD (ng/mL)	LOQ (ng/mL)
Colistin	Y = 5E+06X – 171315	0.9993	0.7	2.2
Melatonin	Y = 3E+07X – 9E+06	0.9823	0.15	0.5

LOD, limit of detection; LOQ, limit of quantity.

Table S7 Recovery, accuracy and precision of colistin and melatonin in plasma samples by LC-MS/MS.

Compounds	Spiked level (ng/mL)	Recovery (%)	Intra-RSD (%) (n = 6)
Colistin	2.2	95.13	3.2
	100	86.57	3.5
	1000	85.36	7.0
Melatonin	0.5	94.12	7.4
	100	87.95	2.3
	1000	92.71	4.3

RSD, relative standard deviation.
